# Nanocellulose Xerogel as Template for Transparent, Thick, Flame-Retardant Polymer Nanocomposites

**DOI:** 10.3390/nano11113032

**Published:** 2021-11-12

**Authors:** Wataru Sakuma, Shuji Fujisawa, Lars A. Berglund, Tsuguyuki Saito

**Affiliations:** 1 Department of Biomaterial Sciences, Graduate School of Agricultural and Life Sciences, The University of Tokyo, 1-1-1 Yayoi, Bunkyo-ku, Tokyo 113-8657, Japan; afujisawa@g.ecc.u-tokyo.ac.jp; 2 Department of Fibre and Polymer Technology, Wallenberg Wood Science Center, KTH Royal Institute of Technology, SE-100 44 Stockholm, Sweden; blund@kth.se

**Keywords:** cellulose nanofibers, nanocomposite, xerogel, flame-retardant

## Abstract

Cellulose nanofibers (CNFs) have excellent properties, such as high strength, high specific surface areas (SSA), and low coefficients of thermal expansion (CTE), making them a promising candidate for bio-based reinforcing fillers of polymers. A challenge in the field of CNF-reinforced composite research is to produce strong and transparent CNF/polymer composites that are sufficiently thick for use as load-bearing structural materials. In this study, we successfully prepared millimeter-thick, transparent CNF/polymer composites using CNF xerogels, with high porosity (~70%) and high SSA (~350 m^2^ g^−1^), as a template for monomer impregnation. A methacrylate was used as the monomer and was cured by UV irradiation after impregnation into the CNF xerogels. The CNF xerogels effectively reinforced the methacrylate polymer matrix, resulting in an improvement in the flexural modulus (up to 546%) and a reduction in the CTE value (up to 78%) while maintaining the optical transparency of the matrix polymer. Interestingly, the composites exhibited flame retardancy at high CNF loading. These unique features highlight the applicability of CNF xerogels as a reinforcing template for producing multifunctional and load-bearing polymer composites.

## 1. Introduction

Polymers reinforced with stiff fillers are candidates for use as low-density structural materials. The effect of reinforcement can be enhanced by nanoscale reinforcement with a large interfacial area between the polymer matrix and reinforcement [1]. Cellulose nanofibers (CNF) are representative nanoscale bio-based reinforcements with excellent mechanical properties and thermal dimensional stability [2]. Therefore, the production of CNF composites with various polymer matrices through diverse preparation processes has been explored in past research studies [2,3,4].

To maximize the potential of CNF composites as structural materials, the following two points are required: (1) high CNF content in the polymer matrix without aggregation, and (2) sufficient thickness, to enable support of high loads. In the field of cellulose nanofiber science, solvent casting is commonly used for the preparation of CNF composites. This process offers transparent, homogeneous, and high-CNF-content polymer composites [4,5]. However, the resulting composites are typically in the form of thin films with thicknesses of around 100 μm or less. To overcome this drawback in solvent casting, the lamination of such thin films using polymers has been proposed [6]. Although the laminated composites were highly transparent and had excellent mechanical properties, the mechanical properties decreased as the number of laminated films increased. Another method of preparing CNF composites is melt compounding, which is practically used and is scalable [7]. Despite these advantages, the addition of CNF leads to increases in the viscosity of the molten polymer, which have been an obstacle to achieving a high CNF content [8]. In addition, in the melt-compounding process, the low dispersibility of CNFs in molten polymers has to be overcome. To improve their dispersibility, CNFs were commonly subjected to surface modification to alter their hydrophilic nature to hydrophobic. However, this modification often decreased the strength of the CNF network by prohibiting strong interactions between the CNFs [9].

One solution to these problems is to impregnate the monomer into the CNF network, followed by curing of the monomer [4,8,10,11,12,13]. Through this method, CNF-rich composites with a highly dispersed CNF network can be obtained without surface modification. In previous studies, thin CNF sheets or delignified wood structures were used as CNF networks [14,15]. The former, being thin materials, cannot be used as structural materials, whereas the latter have a low specific surface area (SSA) and thus cannot maximize the potential of CNFs.

Recently, we developed optically transmissive mesoporous CNF xerogels with high porosity (70–80%) and high SSA (>350 m^2^ g^−1^) [16,17]. Xerogels are porous materials produced through the ambient pressure drying of wet gels. Due to this scalable drying process, xerogels with thicknesses of several millimeters were obtained. Moreover, the CNF xerogels combined high stiffness and high SSA, making them ideal for use as a template for preparing strong and transparent CNF composites.

This study was thus aimed at preparing strong, transparent, thick CNF-rich polymer composites from CNF xerogels using an impregnation method. The CNF content in the composite was varied within a range of 30–80 vol% via the simple densification of CNF xerogels prior to monomer impregnation.

## 2. Materials and Methods

### 2.1. Materials and Chemicals

TEMPO-oxidized pulp with a carboxylate content of approximately 1.8 mmol g^−1^, which was kindly provided by DKS Co. Ltd., (Kyoto, Japan) was used as the starting material for the CNFs (see Figure S1a for Fourier transform infrared (FTIR) spectroscopy analysis) [18]. Aluminum chloride hexahydrate was purchased from FUJIFILM Wako Pure Chemical Corporation (Osaka, Japan). 1-hydroxycyclohexyl phenyl ketone (UV-sensitive radical initiator) and 1,6-hexanediol dimethacrylate were purchased from Tokyo Chemical Industry Co., Ltd. (Tokyo, Japan). All chemicals were used as received.

### 2.2. Preparation of CNF Xerogels

**Figure 1:**
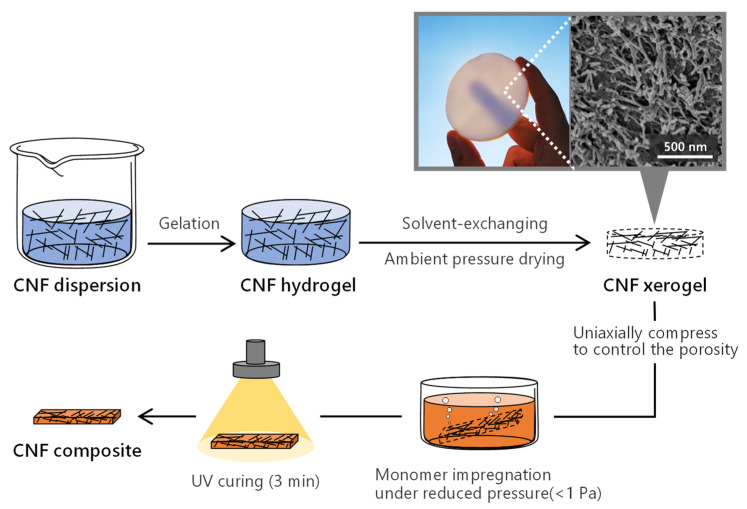
Scheme of CNF composite preparation.

The CNF xerogel was prepared in accordance with our previously reported procedure (Figure 1) [16,17]. A CNF water dispersion was prepared via mechanical disintegration of the TEMPO-oxidized pulp using a high-pressure water jet system (HJP-25005X, Sugino Machine Limited, Toyama, Japan). The width and length of the dispersed CNFs were approximately 2–3 and 300–500 nm, respectively (see Figure S1b for atomic force microscopy (AFM) image). The dispersion was then concentrated to 1.0 wt%. A 0.1 M AlCl_3_ solution was dropped onto the dispersion to obtain the CNF hydrogels. After the solvent of the hydrogels was exchanged with ethanol and hexane, the wet gels were evaporated under ambient pressure at room temperature. The porosity and SSA of the xerogels were 70% and 350 m^2^ g^−1^, respectively. After the xerogels were processed into a certain dimension via sawing and polishing, the CNF content in the final composites was adjusted to be between 30 and 80 vol% via the uniaxial compression of the xerogels. The xerogels were dried at 70 °C for 3 h under reduced pressure prior to the following impregnation procedures.

### 2.3. Preparation of CNF Composites

The initiator was mixed with the monomer at 0.5 wt% for 10 min. After nitrogen purging of the mixture for 5 min, the xerogels were dipped into the monomer solution and then placed under reduced pressure (<1 Pa) until the bubbles arising from the xerogels disappeared. The monomer-containing xerogels were sandwiched between PET films (250 µm thick) with a silicone rubber spacer, and the set was sandwiched between glass plates. Each side of the specimen was then subjected to UV curing for 90 s (for a total of 3 min per specimen) using a high-pressure UV lamp unit (OPM2-502HQ, Ushio Inc., Tokyo, Japan). A pristine polymer matrix (denoted as 0 vol% CNF) was prepared using the same protocol as that used for the composites. The specimens were conditioned at 23 °C and 50% relative humidity for at least 1 d before use.

### 2.4. Analysis

FTIR spectrum of the CNFs was collected using a FT/IR-6100 (JASCO Corp., Tokyo, Japan). AFM observation of the CNFs was conducted using a MultiMode 8 microscope (Bruker, Billerica, MA, USA) equipped with a NanoScope V controller. Diluted CNF water dispersion (0.0005 wt%) was dropped onto a mica plate, and the dried plate was used for the observation. The total light transmittance of the specimens was measured using a UV-Vis V670 (JASCO Corp., Tokyo, Japan) equipped with a horizontal sampling integrating sphere unit (PIN-757). The haze values of the specimens were calculated according to the ASTM D1003 “Standard Test Method for Haze and Luminous Transmittance of Transparent Plastics” [15,19]. The transmittances of composites with different thicknesses were fitted using the following equation, which describes the transmittance of a material at a certain refractive index and wavelength [20]:

(1) $$T={\left(1-R\right)}^{2}\cdot exp\left(-\alpha x\right),$$

where *R*, *α*, and *x* are the coefficient of reflection, attenuation coefficient, and specimen thickness, respectively. The *α* value represents the intensity of attenuation; the higher the *α*, the more intense the attenuation as the thickness increases. The fitting was carried out with varying values of *R* and *α*. A three-point bending test was performed using a EZ-SX (Shimadzu Corp., Kyoto, Japan) equipped with a 500 N load cell. The dimensions of the specimens, span, and crosshead speed were set to 1 × 5 × 30 mm^3^, 20 mm, and 5 mm min^−1^, respectively. Cross-sectional scanning electron microscopy (SEM) observations of the composites were performed using a S-4800 field emission microscope (Hitachi Ltd., Tokyo, Japan) at 1 kV. The specimens were pretreated with a Neo Osmium Coater (Meiwafosis Co., Ltd., Tokyo, Japan) at 5 mA for 10 s. X-ray diffraction measurements were conducted using a MicroMax-007 HF (Rigaku Corp., Tokyo, Japan). The degree of CNF orientation was calculated from the azimuthal profile of 200 reflections [21]. The thermal expansion behaviors of the composites were analyzed using a TMA-60 (Shimadzu Corp., Kyoto, Japan) with a load of 0.03 N and a heating ratio of 2 °C min^−1^ under a nitrogen atmosphere. The dimensions of the specimens and the span were set to 1 × 5 × 12 mm^3^ and 10 mm, respectively. Before measurement, the specimens were dried in the instrument at 120 °C for 90 min. The coefficients of thermal expansion (CTE) of the composites at 30–120 °C were calculated for the thermal expansion curves. The thermal degradation of the composite was investigated using a TA-50 (Shimadzu Corp., Kyoto, Japan) at a heating rate of 10 °C min^−1^ up to 500 °C under a nitrogen or artificial air atmosphere. The starting points of the thermogravimetric (TG) curves were shifted to the value at 100 °C to ignore the weight loss due to humidity in the composites. To investigate the flame retardancy of the composites, the edges of the specimens were exposed to a flame for 3 s and then for 6 s. The dimensions of the specimens were 1 × 5 × 20 mm^3^. All experiments were conducted at least 3 times for each condition.

## 3. Results and Discussion

### 3.1. Preparation of CNF Composite

**Figure 2:**
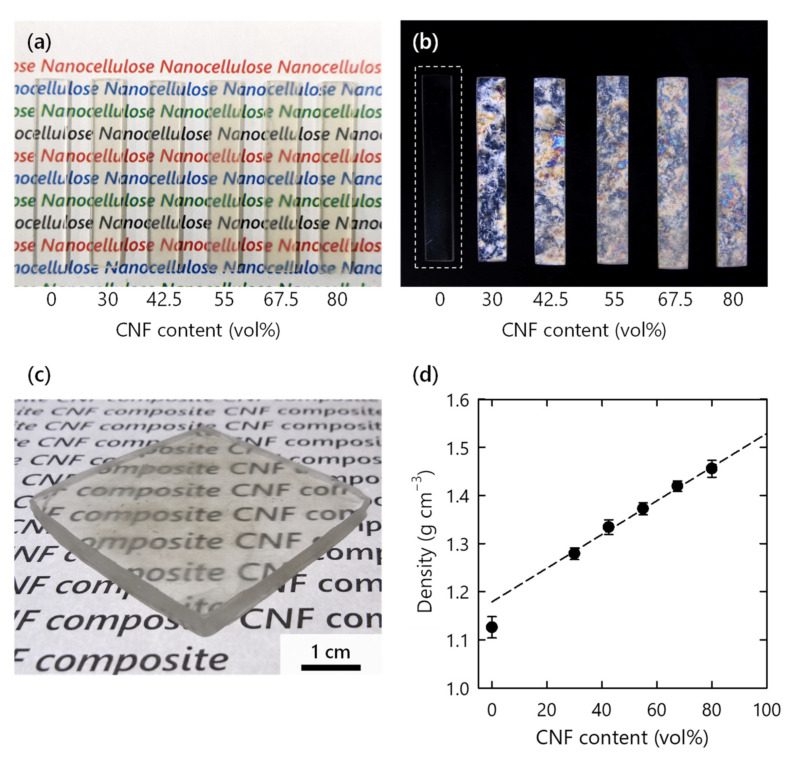
(**a**) Appearance of CNF composites (1 mm thickness); (**b**) appearance of composites observed between crossed polarizers; (**c**) large CNF composite (30 vol%) with 4 mm thickness; (**d**) densities of composites with different CNF contents.

Translucent CNF xerogels became highly transparent after impregnation and curing of the monomer (Figure 2a and Figure S1c). The match of reflective indices between the polymer and CNFs suppressed light scattering [15]. All of the CNF composites exhibited clear birefringence when observed between crossed polarizers, because of the optical anisotropy of the CNFs (Figure 2b) [22]; crystalline CNFs were present throughout the amorphous polymer matrix. The scalable preparation process of the xerogels enabled the production of a thick composite (Figure 2c). To the best of our knowledge, this is the first ever successful one-shot production of a several-millimeter-thick transparent CNF-reinforced polymer composite. The density of the composite exhibited a roughly linear dependence on the CNF content, but the value of the pristine polymer was slightly lower than the extrapolated value at 0 vol% (Figure 2d). This result suggests that the density of the polymer matrix varied based on the presence or absence of a CNF network. The extrapolated value at 100 vol% was also slightly lower than the density of the CNF skeleton (1.58 g cm^−3^). Therefore, limited air voids of up to 9 vol% is one possibility, or increased free volume in the polymer matrix.

### 3.2. Optical Properties

**Figure 3:**
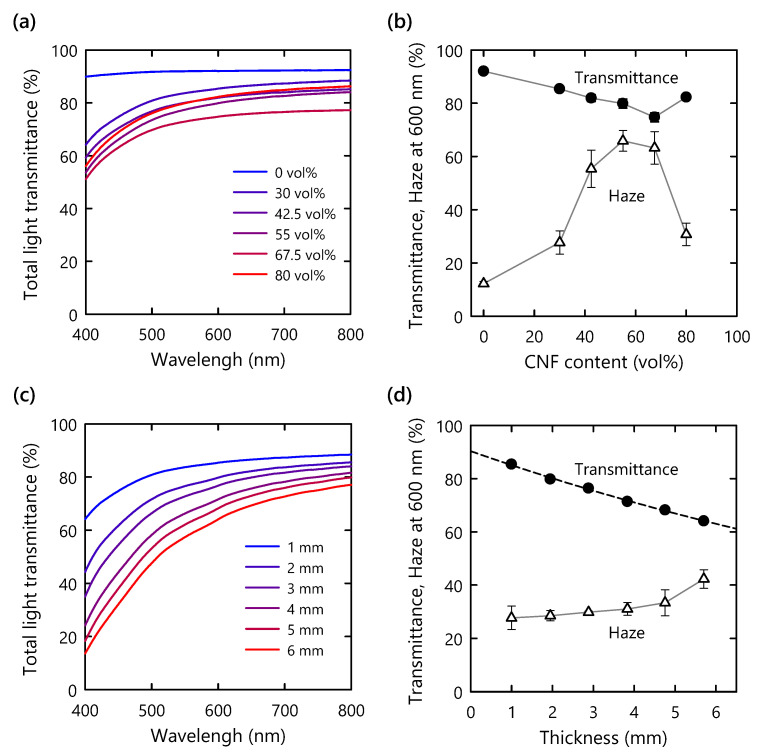
(**a**) Total light transmittance spectra of CNF composites with different CNF contents at 1 mm thickness; (**b**) transmittance and haze values at wavelength of 600 nm as a function of CNF content, same thickness; (**c**) total light transmittance spectra of composites (30 vol%) with different thicknesses; (**d**) transmittance and haze values at wavelength of 600 nm as functions of specimen thickness.

The optical properties of the composite were investigated using UV-Vis spectroscopy. As mentioned in the previous section, all the composites showed high transparencies in the visible-light range, regardless of the CNF content (Figure 3a). At a CNF content of 50–70 vol%, the total transmittance and haze value at a wavelength of 600 nm exhibited their lowest and highest values, respectively (Figure 3b). This phenomenon is likely explained by light scattering at the interface between the polymer matrix and the CNF. The number of scattering interfaces should increase as the CNF content increases. However, over a certain threshold of the CNF content, CNFs should start to contact with one another. Accordingly, the scattering interfaces between the polymer matrix and the CNFs are supposed to decrease at such a high CNF content [23]. Therefore, we assumed that the number of scattering interfaces reached its maximum value at a CNF content of 50–70 vol% and then decreased in the CNF content from 70 to 80 vol%, resulting in an increase in transmittance and a decrease in haze value. It should be emphasized that the reduction in transmittance relative to that of the pristine polymer matrix was only 10% for the composite with a high CNF content of 80 vol% and a thickness of 1 mm.

**Figure 4:**
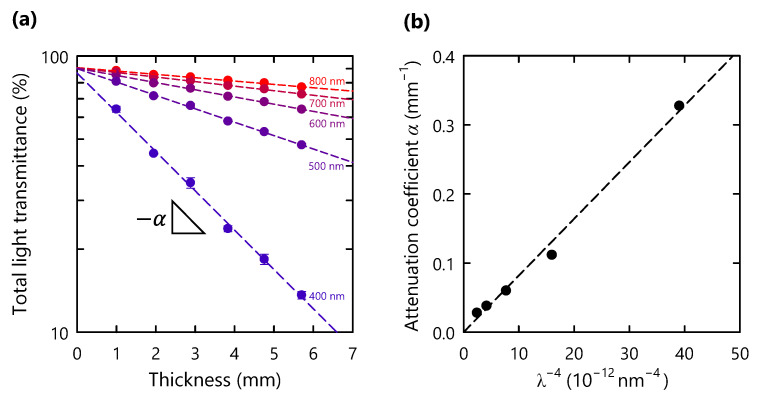
(**a**) Total light transmittance of CNF composites (30 vol%) with different specimen thicknesses at different wavelengths; (**b**) attenuation coefficient (*α*) plot as function of inverse fourth power of wavelength (*λ*^−4^).

To examine the relationship of the optical properties with the thickness, 30 vol% CNF composites with different thicknesses were prepared. As the thickness was increased, the total transmittance and haze value of the composites gradually decreased and increased, respectively (Figure 3c,d). The transmittances at different wavelengths were fitted using Equation (1) (Figure 4a). At a wavelength of 600 nm, the *α* value in Equation (1) was calculated to be 0.06 mm^−1^, which is lower than the previously reported value for a delignified-wood-based polymer composite without surface modification (0.16 mm^−1^) [24]. The low dependence of the optical properties on thickness implies the absence of micron-sized voids in the composite and the favorable match in refractive index between the polymer and the CNFs [24].

The *α* value has a proportional relationship with the number of scattering centers per unit volume (*N*), the radius of scattering centers (*r*), and wavelength (*λ*) as follows [20]:

(2) $$\alpha =A\cdot N\cdot r^{6}\cdot \lambda^{-4},$$

where *A* is a constant. This simplified equation for Rayleigh scattering involves the following assumptions: (1) negligible light absorption; (2) the constant refractive index of the polymer and CNF as a function of wavelength; and (3) a much smaller *r* value compared to the wavelength. Interestingly, the *α* values of our composites exhibited a nearly linear dependence on *λ*^−4^ in the wavelength range of 400–800 nm (Figure 4b). Therefore, the number of scattering centers per unit volume (*N*) and radius (*r*) of centers (i.e., CNFs in the composite) are constants in Equation (2). These results indicate that the CNF composites have a highly homogeneous structure throughout the material with a thickness of up to 6 mm.

### 3.3. Mechanical Properties

**Figure 5:**
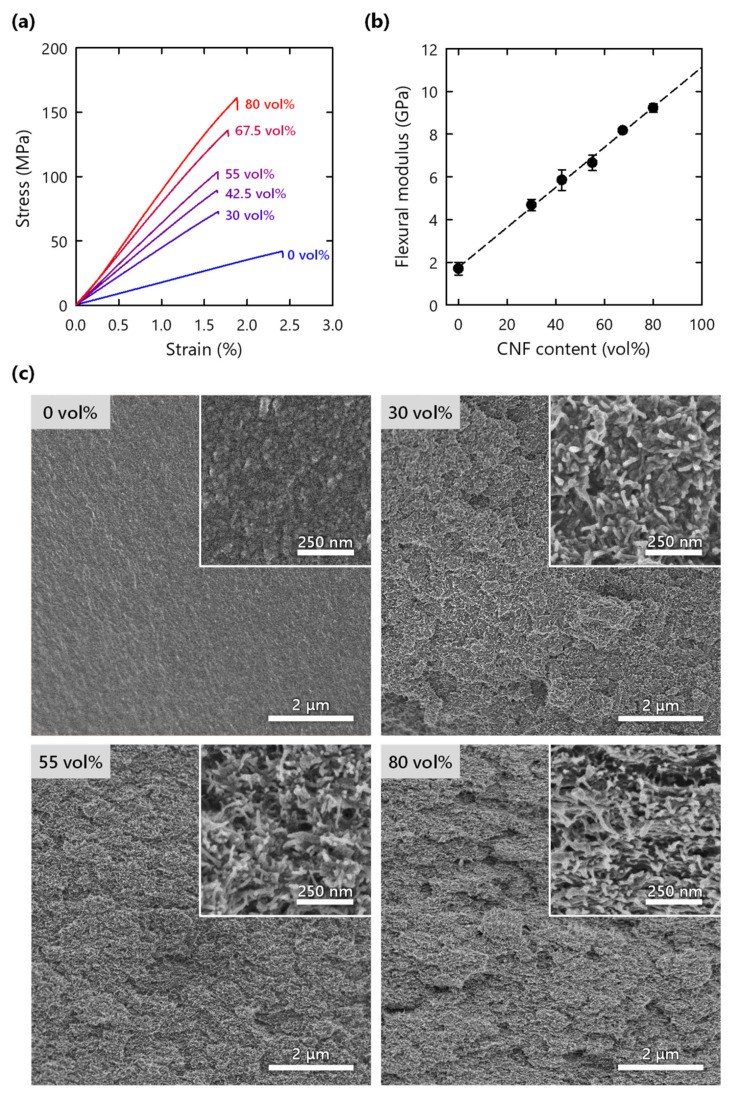
(**a**) Stress–strain curves estimated from three-point bending test of CNF composites; (**b**) flexural modulus of composite as function of CNF content; (**c**) SEM images of fractured surfaces of composites.

The mechanical properties of the composites were evaluated using a three-point bending test. The CNF network in the polymer exhibited a significant reinforcement effect (Figure 5a). The addition of 30 vol% CNF doubled the modulus and strength of the pristine polymer. Furthermore, the composite with 80 vol% CNF reached a strength of 160 MPa, which is comparable to those of glasses [25]. The strain at failure of the composite was slightly lower than that of the pristine polymer, although the matrix also has low strain to failure. The moduli of the composites exhibited a linear relationship with CNF content (Figure 5b). A simple mixing model was used [26]:

(3) $$E_{c}=E_{\mathrm{np}}V_{f}+E_{m}(1-V_{f})$$

where *E*_c_, *E*_m_, and *E*_np_ are the elastic moduli of the composite, polymer matrix, and neat CNF nanopaper, respectively, and *V*_f_ is the volume fraction of CNF (note that the CNF nanopaper refers to a film obtained through the evaporative drying of CNF water dispersion). *E*_np_, or effective reinforcement modulus, was set to be a free parameter and estimated from linear regression of the experimental data. The obtained *E*_np_ for the reinforcement phase, 11.3 GPa, was within the range of that of neat, dense CNF networks (9–15 GPa) [21,26], suggesting that the network structure of the xerogel has a dominant role in the stiffness of the composites [10,26]. One of the challenges in the field of CNF composites is the decrease in the reinforcement efficiency at high fiber content due to CNF aggregation [8]. This study avoided critical aggregation and achieved a linear reinforcement effect in a wide range of CNF content. The values of flexural strength and work of fracture (area under stress–strain curve) were very scattered, probably because of technical problems in the composite preparation, such as voids in the composites (Figure S2a,b). Nonetheless, improvements in the strength and work of fracture due to CNF addition were still confirmed.

Through SEM observations of the fractured surface, a macroscopically rough structure was confirmed for the CNF composite, whereas a smooth fracture surface was observed for the pristine polymer (Figure 5c and Figure S2c). These morphologies suggest more complex crack propagation due to the CNFs [27,28]. This consumes more energy and may contribute to an increase in work of fracture. A homogeneous CNF network was observed in the composites even at a high CNF content, without any sign of layered structures (inset of Figure 5c and Figure S2c). In addition, the individual fibers appear to be uncovered by the polymer, implying pull-out of the CNFs although the lengths are short.

These results indicate that the mechanical properties of the composites were governed by the CNF reinforcement network.

### 3.4. Thermal Expansion Behavior

**Figure 6:**
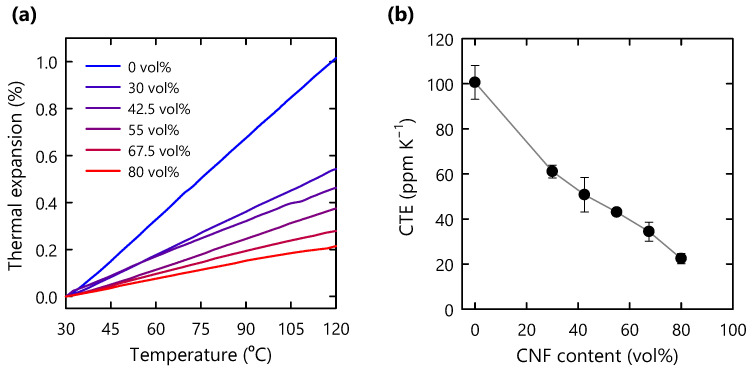
(**a**) Thermal expansion behavior of CNF composites; (**b**) CTE values of composites as functions of CNF content.

In the case of thick and large structural materials, dimensional changes due to thermal expansion are more pronounced. In this study, the nanoscale CNF network successfully restricted the expansion of the polymer matrix in thick composites (Figure 6a). The CTE values, which were calculated from the slopes of the thermal expansion curves, decreased as the CNF content increased (Figure 6b). The composite with 80 vol% CNF exhibited a 78% reduction in the CTE value with respect to that of the pristine polymer. Meanwhile, a number of previous studies on thin-film CNF composites with low CNF content reported comparable or even lower CTE values [5,29,30,31]. Such low CTE values for thin-film composites may have been achieved through strong in-plane orientations of CNFs in the composites [32,33]. By comparison, in this study, the degree of CNF in-plane orientation in the composite was not as high as those of thin-film composites (Figure S3).

### 3.5. Flame Retardancy

**Figure 7:**
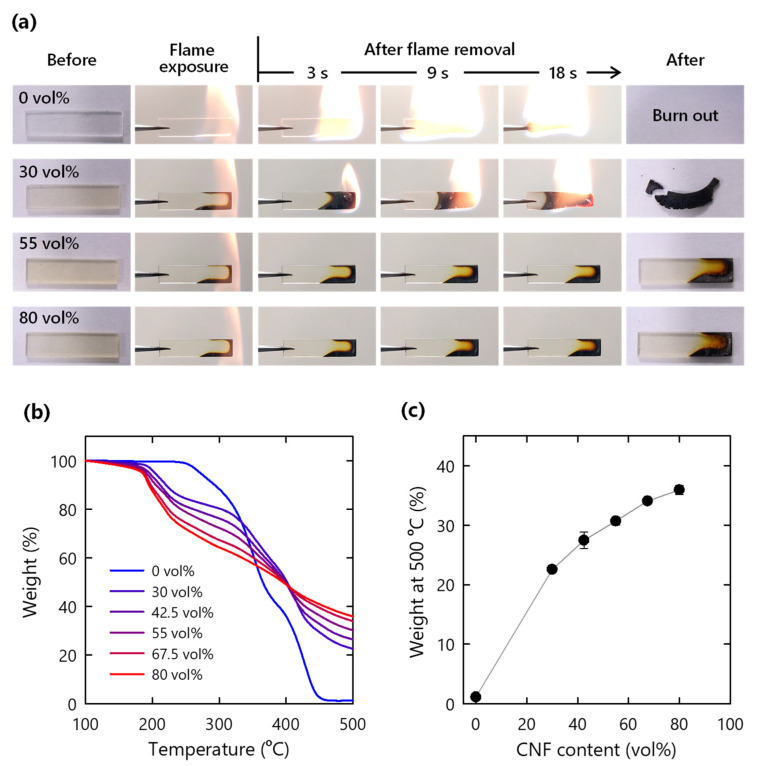
(**a**) Flammability test of CNF composites; (**b**) TG curves of CNF composites under nitrogen conditions; (**c**) residue weight at 500 °C based on TG analysis under nitrogen conditions.

The CNF xerogels used in this study have a flame self-extinguishing functionality [17]. Thus, the composites were expected to inherit a similar functionality. After being exposed to flame for 3 s, the pristine polymer was rapidly and completely consumed by the flame (Figure 7a and Video S1). By contrast, the CNF composite with 30–42.5% CNF burned slowly, and a black residue remained (Figure 7a and Figure S4a, and Video S2). Interestingly, for the composites containing more than 55 vol% CNF, no flame ignition was observed (Figure 7a and Figure S4a, and Video S3). The non-combusted specimens were further exposed to a flame for 6 s. For the composites with 67.5% and 80 vol% CNF, the ignited flame was self-extinguished (Figure S4b and Video S4).

Figure 7b shows the TG curves under nitrogen conditions. The CNF composites started to degrade at approximately 200 °C prior to polymer degradation at 250 °C (Figure 7b). The initial thermal degradation of the composite was derived based on the decarboxylation of TEMPO-oxidized cellulose [34,35]. The non-flammable carbon dioxide emitted from cellulose possibly dilutes the flammable gases [36,37]. The residue weight at 500 °C increased as the CNF content increased (Figure 7c). In contrast, the pristine polymer completely degraded to volatile gas at 450 °C; the CNF composite with 80 vol% CNF retained 35% of its weight at 500 °C. The increase in the residual weight is likely explained by the thermally stable char formation promoted by metal ions on the cellulose fiber surface [35,36,37,38]. In addition, aluminum hydroxide structure on the CNF surface dehydrates into aluminum oxide via an endothermic reaction during flame exposure [17]. The formed involatile residues, including char and aluminum oxide, should contribute to the flame retardancy of the CNF composites [36,37]. Meanwhile, the TG curves under air conditions demonstrated similar trends to those under nitrogen conditions (Figure S4c,d). This indicates that oxidation is suppressed, possibly because of barrier function of CNF and CNF char.

## 4. Conclusions

In this study, thick CNF/polymer composites were prepared via an impregnation method using nanocellulose xerogels. The composite exhibited high optical transmittance over a broad range of CNF content. Analysis of the relationship of the transmittance with thickness suggested that the composite has a homogeneous structure with well-dispersed CNF fibrils. The fine CNF network efficiently reinforced the polymer matrix, resulting in improvements in modulus, strength, and work of fracture. The network also contributed to the thermal stability of the composite, with a reduction in the CTE value of up to 78%. Additionally, the wood-derived nanofibers endowed the composite with flame retardancy. These unique features highlight the applicability of CNF xerogels as a reinforcing template for producing multifunctional and load-bearing polymer composites.

## Data Availability

The data that support the findings of this study are available from the corresponding authors upon reasonable request.

## Supplementary Materials

The following are available online at https://www.mdpi.com/article/10.3390/nano11113032/s1, Figure S1: FTIR spectrum and AFM height image of the CNFs, and appearance of CNF xerogels, Figure S2: Flexural strength, work of fracture, and fractured surfaces of CNF composites, Figure S3: X-ray diffraction patterns of composites, Figure S4: Flammability test for CNF composites and TG data under air conditions, Video S1: Flammability test for pristine polymer, Video S2: Flammability test for CNF composite (30 vol%), Video S3: Flammability test for CNF composite (55 vol%), Video S4: Additional flammability test for CNF composite (80 vol%).

## References

[1] Harito C., Bavykin D.V., Yuliarto B., Dipojono H.K., Walsh F.C. (2019). Polymer Nanocomposites Having a High Filler Content: Synthesis, Structures, Properties, and Applications. Nanoscale.

[2] Moon R.J., Martini A., Nairn J., Simonsen J., Youngblood J. (2011). Cellulose Nanomaterials Review: Structure, Properties and Nanocomposites. Chem. Soc. Rev..

[3] Clarkson C.M., El Awad Azrak S.M., Forti E.S., Schueneman G.T., Moon R.J., Youngblood J.P. (2020). Recent Developments in Cellulose Nanomaterial Composites. Adv. Mater..

[4] Oksman K., Aitomäki Y., Mathew A.P., Siqueira G., Zhou Q., Butylina S., Tanpichai S., Zhou X., Hooshmand S. (2016). Review of the Recent Developments in Cellulose Nanocomposite Processing. Compos. Part A Appl. Sci. Manuf..

[5] Fujisawa S., Togawa E., Kimura S. (2018). Large Specific Surface Area and Rigid Network of Nanocellulose Govern the Thermal Stability of Polymers: Mechanisms of Enhanced Thermomechanical Properties for Nanocellulose/PMMA Nanocomposite. Mater. Today Commun..

[6] Forti E.S., Moon R.J., Schueneman G.T., Youngblood J.P. (2020). Transparent Tempo Oxidized Cellulose Nanofibril (TOCNF) Composites with Increased Toughness and Thickness by Lamination. Cellulose.

[7] Hietala M., Mathew A.P., Oksman K. (2013). Bionanocomposites of Thermoplastic Starch and Cellulose Nanofibers Manufactured Using Twin-Screw Extrusion. Eur. Polym. J..

[8] Ansari F., Berglund L.A. (2018). Toward Semistructural Cellulose Nanocomposites: The Need for Scalable Processing and Interface Tailoring. Biomacromolecules.

[9] Dufresne A. (2017). Cellulose Nanomaterial Reinforced Polymer Nanocomposites. Curr. Opin. Colloid Interface Sci..

[10] Capadona J.R., Van Den Berg O., Capadona L.A., Schroeter M., Rowan S.J., Tyler D.J., Weder C. (2007). A Versatile Approach for the Processing of Polymer Nanocomposites with Self-Assembled Nanofibre Templates. Nat. Nanotechnol..

[11] Yano H., Sugiyama J., Nakagaito A.N., Nogi M., Matsuura T., Hikita M., Handa K. (2005). Optically Transparent Composites Reinforced with Networks of Bacterial Nanofibers. Adv. Mater..

[12] Ansari F., Skrifvars M., Berglund L. (2015). Nanostructured Biocomposites Based on Unsaturated Polyester Resin and a Cellulose Nanofiber Network. Compos. Sci. Technol..

[13] Nakagaito A.N., Yano H. (2005). Novel High-Strength Biocomposites Based on Microfibrillated Cellulose Having Nano-Order-Unit Web-like Network Structure. Appl. Phys. A Mater. Sci. Process..

[14] Iwamoto S., Nakagaito A.N., Yano H., Nogi M. (2005). Optically Transparent Composites Reinforced with Plant Fiber-Based Nanofibers. Appl. Phys. A Mater. Sci. Process..

[15] Li Y., Fu Q., Yu S., Yan M., Berglund L. (2016). Optically Transparent Wood from a Nanoporous Cellulosic Template: Combining Functional and Structural Performance. Biomacromolecules.

[16] Yamasaki S., Sakuma W., Yasui H., Daicho K., Saito T., Fujisawa S., Isogai A., Kanamori K. (2019). Nanocellulose Xerogels With High Porosities and Large Specific Surface Areas. Front. Chem..

[17] Sakuma W., Yamasaki S., Fujisawa S., Kodama T., Shiomi J., Kanamori K., Saito T. (2021). Mechanically Strong, Scalable, Mesoporous Xerogels of Nanocellulose Featuring Light Permeability, Thermal Insulation, and Flame Self-Extinction. ACS Nano.

[18] Saito T., Nishiyama Y., Putaux J.L., Vignon M., Isogai A. (2006). Homogeneous Suspensions of Individualized Microfibrils from TEMPO-Catalyzed Oxidation of Native Cellulose. Biomacromolecules.

[19] ASTM D1003-21 (2021). Standard Test Method for Haze and Luminous Transmittance of Transparent Plastics.

[20] Faure B., Salazar-Alvarez G., Ahniyaz A., Villaluenga I., Berriozabal G., De Miguel Y.R., Bergström L. (2013). Dispersion and Surface Functionalization of Oxide Nanoparticles for Transparent Photocatalytic and UV-Protecting Coatings and Sunscreens. Sci. Technol. Adv. Mater..

[21] Zhao M., Ansari F., Takeuchi M., Shimizu M., Saito T., Berglund L.A., Isogai A. (2018). Nematic Structuring of Transparent and Multifunctional Nanocellulose Papers. Nanoscale Horiz..

[22] Saito T., Uematsu T., Kimura S., Enomae T., Isogai A. (2011). Self-Aligned Integration of Native Cellulose Nanofibrils towards Producing Diverse Bulk Materials. Soft Matter.

[23] Parlak O., Demir M.M. (2013). Anomalous Transmittance of Polystyrene–Ceria Nanocomposites at High Particle Loadings. J. Mater. Chem. C.

[24] Chen H., Baitenov A., Li Y., Vasileva E., Popov S., Sychugov I., Yan M., Berglund L. (2019). Thickness Dependence of Optical Transmittance of Transparent Wood: Chemical Modification Effects. ACS Appl. Mater. Interfaces.

[25] Sharma B., Gatóo A., Bock M., Ramage M. (2015). Engineered Bamboo for Structural Applications. Constr. Build. Mater..

[26] Lee K.Y., Aitomäki Y., Berglund L.A., Oksman K., Bismarck A. (2014). On the Use of Nanocellulose as Reinforcement in Polymer Matrix Composites. Compos. Sci. Technol..

[27] Shir Mohammadi M., Hammerquist C., Simonsen J., Nairn J.A. (2016). The Fracture Toughness of Polymer Cellulose Nanocomposites Using the Essential Work of Fracture Method. J. Mater. Sci..

[28] Gao H., Qiang T. (2017). Fracture Surface Morphology and Impact Strength of Cellulose/PLA Composites. Materials.

[29] Nogi M., Ifuku S., Abe K., Handa K., Nakagaito A.N., Yano H. (2006). Fiber-Content Dependency of the Optical Transparency and Thermal Expansion of Bacterial Nanofiber Reinforced Composites. Appl. Phys. Lett..

[30] Okahisa Y., Yoshida A., Miyaguchi S., Yano H. (2009). Optically Transparent Wood–Cellulose Nanocomposite as a Base Substrate for Flexible Organic Light-Emitting Diode Displays. Compos. Sci. Technol..

[31] Nakagaito A.N., Yano H. (2008). The Effect of Fiber Content on the Mechanical and Thermal Expansion Properties of Biocomposites Based on Microfibrillated Cellulose. Cellulose.

[32] Diaz J.A., Wu X., Martini A., Youngblood J.P., Moon R.J. (2013). Thermal Expansion of Self-Organized and Shear-Oriented Cellulose Nanocrystal Films. Biomacromolecules.

[33] Hirano T., Mitsuzawa K., Ishioka S., Daicho K., Soeta H., Zhao M., Takeda M., Takai Y., Fujisawa S., Saito T. (2020). Anisotropic Thermal Expansion of Transparent Cellulose Nanopapers. Front. Chem..

[34] Fukuzumi H., Saito T., Okita Y., Isogai A. (2010). Thermal Stabilization of TEMPO-Oxidized Cellulose. Polym. Degrad. Stab..

[35] Lichtenstein K., Lavoine N. (2017). Toward a Deeper Understanding of the Thermal Degradation Mechanism of Nanocellulose. Polym. Degrad. Stab..

[36] Geng C., Zhao Z., Xue Z., Xu P., Xia Y. (2019). Preparation of Ion-Exchanged TEMPO-Oxidized Celluloses as Flame Retardant Products. Molecules.

[37] Guo X., Wang Y., Ren Y., Liu X. (2021). Fabrication of Flame Retardant Lyocell Fibers Based on Carboxymethylation and Aluminum Ion Chelation. Cellulose.

[38] Shi R., Tan L., Zong L., Ji Q., Li X., Zhang K., Cheng L., Xia Y. (2017). Influence of Na+ and Ca2+ on Flame Retardancy, Thermal Degradation, and Pyrolysis Behavior of Cellulose Fibers. Carbohydr. Polym..

