# Supplementary material for: Nanocellulose Xerogel as Template for Transparent, Thick, Flame-Retardant Polymer Nanocomposites
Source: Nanomaterials (Basel). 2021 Nov 12;11(11):3032. doi: 10.3390/nano11113032 (PMC8619435; doi:10.3390/nano11113032)
Supplement: Supplementary file 1 [file nanomaterials-11-03032-s001.zip › nanomaterials-1451573-SI/nanomaterials-1451573-SI.pdf]

nanomaterials

MDPI

Supplementary Materials

Nanocellulose Xerogel as Template for Transparent, Thick, Flame-Retardant Polymer Nanocomposites

Wataru Sakuma [1,*], Shuji Fujisawa [1], Lars A. Berglund [2] and Tsuguyuki Saito [1,*]

1 Department of Biomaterial Sciences, Graduate School of Agricultural and Life Sciences, The University of Tokyo, 1-1-1 Yayoi, Bunkyo-ku, Tokyo 113-8657, Japan; afujisawa@g.ecc.u-tokyo.ac.jp
2 Department of Fibre and Polymer Technology, Wallenberg Wood Science Center, KTH Royal Institute of Technology, SE-100 44 Stockholm, Sweden; blund@kth.se
* Correspondence: sakuma.wataru@gmail.com (W.S.), saitot@g.ecc.u-tokyo.ac.jp (T.S.); Tel.: +81-3-5841-5271 (T.S.)

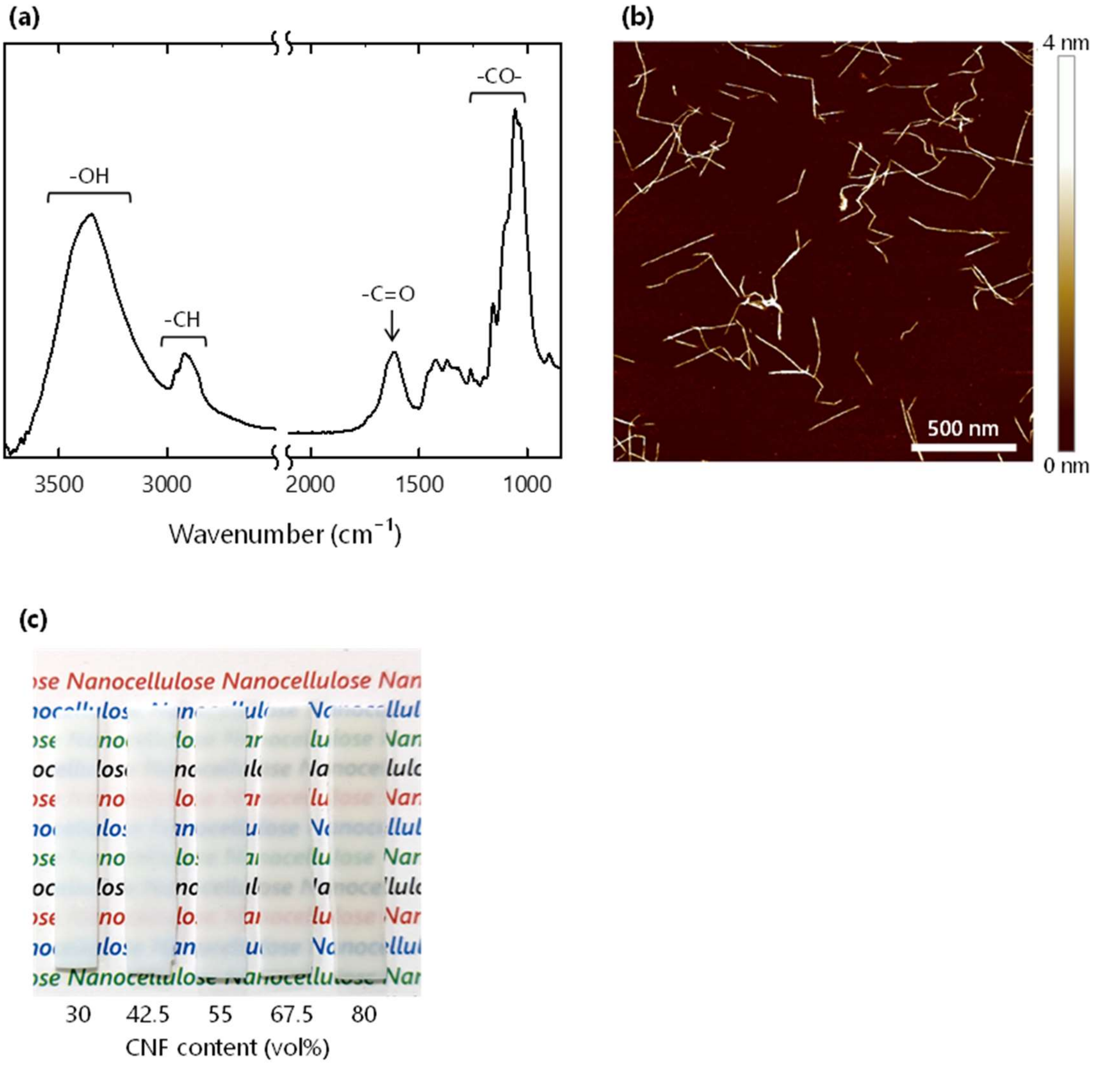

Figure S1. (**a**) FTIR spectrum and (**b**) AFM height image of the CNFs prepared in this study; (**c**) Appearance of the CNF xerogels with different bulk densities (thickness 1 mm). The FTIR spectrum (panel a) shows typical characteristics for TEMPO-oxidized cellulose [18]; the absorption peaks at approximately 3200–3500 cm^{-1}, 2900 cm^{-1}, 1600 cm^{-1} and 1000–1200 cm^{-1} are due to the stretching vibrations of the -OH, -CH, -C=O and -CO- moieties in TEMPO-oxidized cellulose, respectively.

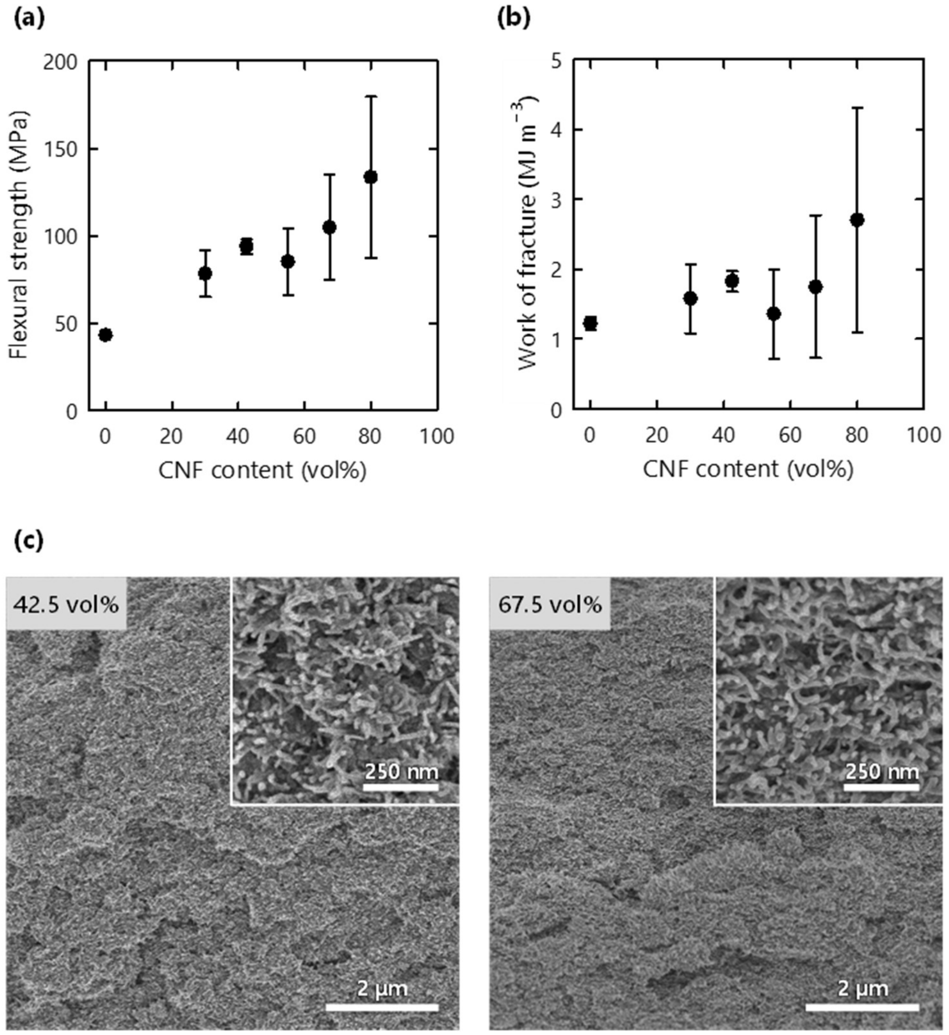

Figure S2. (**a**) Flexural strength and (**b**) work of fracture of composites as functions of CNF content; (**c**) SEM images of fractured surfaces of composites.

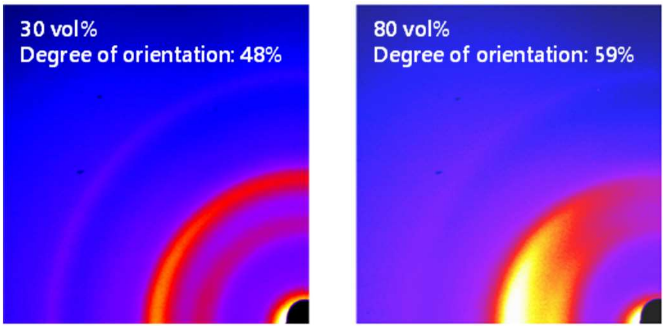

Figure S3. X-ray diffraction patterns of xerogels for incident beam parallel to in plane.

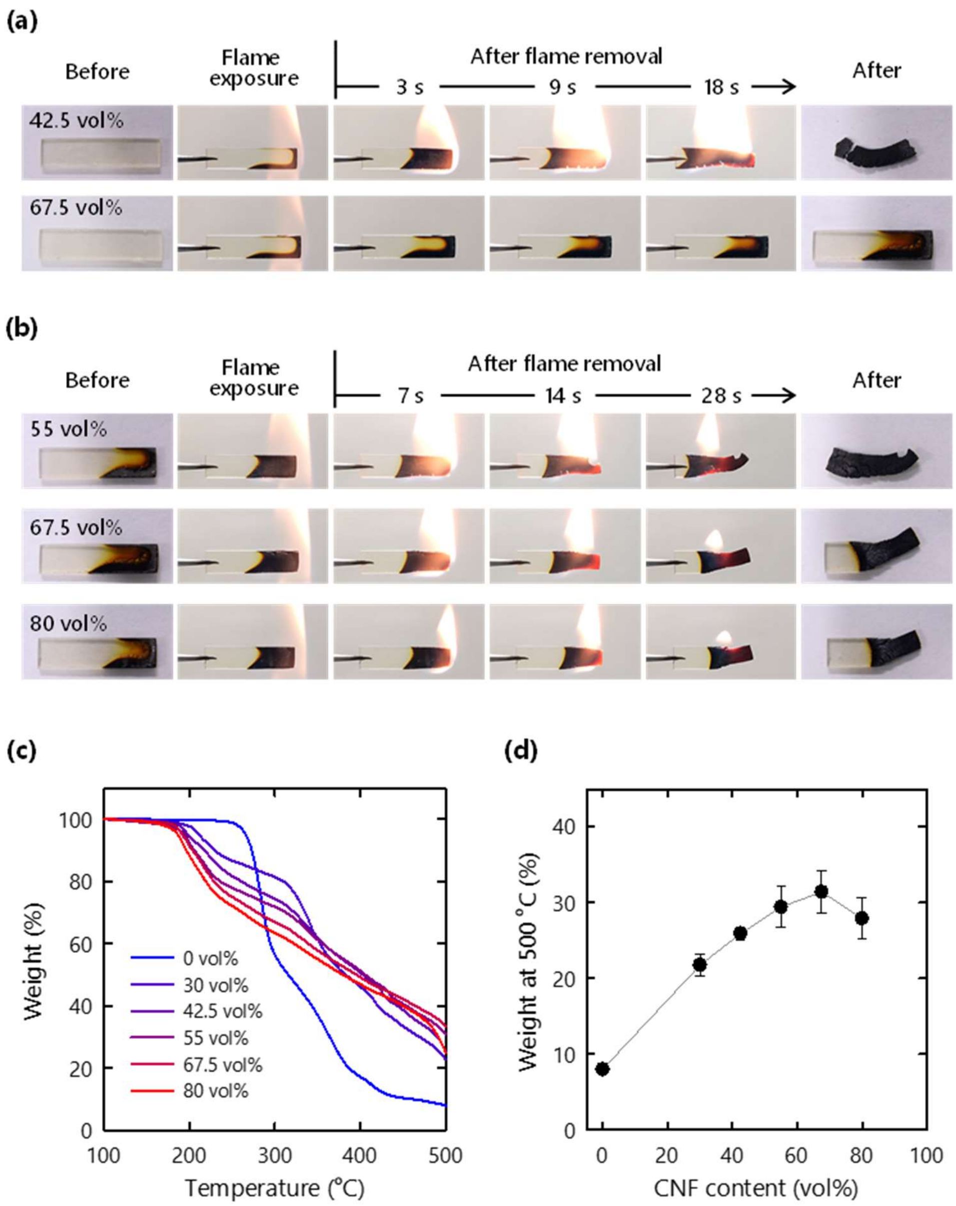

Figure S4. (**a**) Flammability test of CNF composites; (**b**) Additional flammability test of CNF composites with high CNF content; (**c**) TG curves of CNF composites under air conditions; (**d**) Residue weight at 500 °C based on TG analysis under air conditions.
